# Identification of NTRK3 as a potential prognostic biomarker associated with tumor mutation burden and immune infiltration in bladder cancer

**DOI:** 10.1186/s12885-021-08229-1

**Published:** 2021-04-24

**Authors:** Zhao Zhang, Yongbo Yu, Pengfei Zhang, Guofeng Ma, Mingxin Zhang, Ye Liang, Wei Jiao, Haitao Niu

**Affiliations:** 1 Department of Urology, The Affiliated Hospital of Qingdao University, No.16 Jiangsu Road, Qingdao, 266000 China; 2 Key Laboratory, Department of Urology and Andrology, The Affiliated Hospital of Qingdao University, Qingdao, China

**Keywords:** Tumor mutation burden, Immune infiltration, NTRK3, Prognosis, TCGA

## Abstract

**Background:**

Bladder cancer (BLCA) is a common malignant tumor of urinary system with high morbidity and mortality. In recent years, immunotherapy has played a significant role in the treatment of BLCA. Tumor mutation burden (TMB) has been reported to be a powerful biomarker for predicting tumor prognosis and efficacy of immunotherapy. Our study aimed to explore the relationship between TMB, prognosis and immune infiltration to identify the key genes in BLCA.

**Methods:**

Clinical information, somatic mutation and gene expression data of BLCA patients were downloaded from The Cancer Genome Atlas (TCGA) database. Patients were divided into high and low TMB groups according to their calculated TMB scores. Gene Set Enrichment Analysis (GSEA) was performed to screen for significantly enriched pathways. Differentially expressed genes (DEGs) between the two groups were identified. Univariate Cox analysis and Kaplan-Meier survival analysis were applied for screening key genes. Immune infiltration was performed for TMB groups and NTRK3.

**Results:**

Higher TMB scores were related with poor survival in BLCA. After filtering, 36 DEGs were identified. NTRK3 had the highest hazard ratio and significant prognostic value. Co-expressed genes of NTRK3 were mainly involved in several pathways, including DNA replication, basal transcription factors, complement and coagulation cascades, and ribosome biogenesis in eukaryotes. There was a significant correlation among TMB scores, NTRK3 expression and immune infiltration.

**Conclusions:**

Our results suggest that NTRK3 is a TMB-related prognostic biomarker, which lays the foundation for further research on the immunomodulatory effect of NTRK3 in BLCA.

**Supplementary Information:**

The online version contains supplementary material available at 10.1186/s12885-021-08229-1.

## Background

Bladder cancer (BLCA) is one of the most common urinary malignancies and its incidence is gradually increasing, accounting for nearly 550,000 new cases and 200,000 deaths annually worldwide [1]. 75% of newly diagnosed patients are nonmuscle-invasive bladder cancers (NMIBCs), and although the 5-year overall survival (OS) rate is as high as 90% after treatment, NMIBC has a high recurrence rate, presenting a large social and economic burden [2, 3]. Muscle-invasive bladder cancer (MIBC) represents approximately 20% of newly diagnosed cases, and approximately 15 to 20% of NMIBCs can progress to MIBCs [4]. Despite radical cystectomy and neoadjuvant chemotherapy are available, the MIBC patients have a poor prognosis with a 5-year OS rate of less than 50%. In addition, about half of the patients will eventually progress to distant metastases [5, 6].

In the past 30 years, the systemic treatment for BLCA has remained unchanged and the curative effect has not made a breakthrough, especially in advanced disease [7]. Nowadays, with the rapid evolution of immunotherapy, the use of immune checkpoint inhibitors (ICIs) is becoming emerging as a new treatment strategy for advanced BLCA [8]. Treatment with ICIs such as anti-programmed cell death protein 1(PD-1), anti-programmed death-ligand 1 (PD-L1), and anti-cytotoxic T-lymphocyte-associated protein 4 (CTLA-4) can significantly increase OS and result in durable remission for many advanced cancers, including melanoma, non-small cell lung cancer, renal cancer and urothelial cancer [9–12]. However, these effects only exist in a small subset of patients who can respond to ICIs. Many studies have shown that the tumor mutation burden (TMB) is a powerful biomarker for predicting the efficacy of ICIs and can be used to identify patients who will benefit from immunotherapy [13]. Tumors can be caused by the accumulation of somatic mutations in cells as a result of carcinogens. Some mutant cells revert to normal via DNA self-modification, some die, and a minority of them express neoantigens on surface. Tumor cells can prevent these mutant cells from being recognized by the immune system through abnormal expressions of antigens or regulating the tumor microenvironment (TME), thereby achieving immune escape [14]. A higher TMB represents more neoantigens and increases the probability of activating the body’s anti-tumor immune response, which can improve the patients’ response to ICIs [15, 16]. Thus, exploring the relationship between TMB and immunoregulation and discovering the related genes or biological mechanisms will help to better understand the role of immunotherapy in cancer treatment.

In our study, mutation information and expression profiles were acquired from The Cancer Genome Atlas (TCGA) database. Bioinformatic analysis was applied to calculate TMB scores and evaluate their relationships with prognosis and immune infiltration. Finally, we identified one key potential biomarker with prognostic value, which might help to filter BLCA patients suitable for immunotherapy.

## Methods

### Data source and data processing

Transcriptome data and clinical information of BLCA patients were downloaded from the TCGA data portal (http://cancergenome.nih.gov/). Validation cohort GSE48276 was downloaded from the GEO database (https://www.ncbi.nlm.nih.gov/geo/). Then we downloaded the simple nucleotide variation data from the “Masked Somatic Mutation” category processed with VarScan software in TCGA. The “maftools” R package was used to analyze the Mutation Annotation Format (MAF) file and visualize the somatic mutation data [17].

### Calculation of TMB scores and prognostic evaluation

TMB is also defined as mutation frequency. We calculated TMB scores with the number of all nonsynonymous mutations, divided by exon length (38 Mb). We used the X-title software to calculate the best cut-off value of TMB scores and divided the patients into high and low TMB groups [18]. Survival analysis was conducted while clinicopathological characteristics were compared between high and low TMB groups. The Wilcoxon rank-sum test was for comparing two groups and the Kruskal–Wallis test was for more groups.

### Gene set enrichment analysis (GSEA) between high and low TMB groups

GSEA version was 4.0.0 based on JAVA platform and the reference gene set (c2.cp.kegg.v7.0.symbols.gmt) was downloaded from the Molecular Signatures Database (MSigDB) (http://software.broadinstitute.org/gsea/msigdb/) [19]. The pathways were considered to be statistically enriched with *P* < 0.05 and FDR (false discovery rate) < 0.25.

### Screening of key genes associated with TMB and survival

We identified the differentially expressed genes (DEGs) between high and low TMB groups. The “edgeR,” “limma,” and “DEseq2” packages in R software were used for this analysis. Univariate Cox and Kaplan-Meier (KM) methods were performed for survival analysis on every DEG to obtain the target genes. |log_2_FC| > 1 and *P* value < 0.05 were set as the thresholds.

### Coexpression analysis of NTRK3

LinkedOmics (http://www.linkedomics.org/login.php) is a publicly available portal that includes multi-omics data from all 32 TCGA cancer types and provides a web-based platform for analysis [20]. Coexpression of NTRK3 was analyzed statistically on the website using the Pearson correlation coefficient and the results were visualized by volcano plot and heat map. The LinkInterpreter module performed GSEA to obtain the related Kyoto Encyclopedia of Genes and Genomes (KEGG) pathways. The rank criterion was *P* < 0.05 and 500 simulations were tested.

### Immune infiltration analysis

The “estimate” R package was used to calculate scores for tumor purity, the level of stromal cells presents, and the infiltration level of immune cells in tumor tissues based on expression data. The CIBERSORT script was used to analyze 22 types of immune cell fractions in each patient [21]. Samples with a *P* value ≥0.05 were excluded from the next step. Wilcoxon rank-sum tests were conducted to analyze the different abundances of 22 immune cell types between high and low TMB groups. The TIMER database (https://cistrome.shinyapps.io/timer) was used to analyze the correlation between NTRK3 expression and immune cell infiltration level [22]. The TISIDB web portal (http://cis.hku.hk/TISIDB/index.php) was applied to explore the association between NTRK3 and multiple immune regulatory factors [23].

## .Results

### Overview of mutation information in BLCA patients

**Fig. 1:**
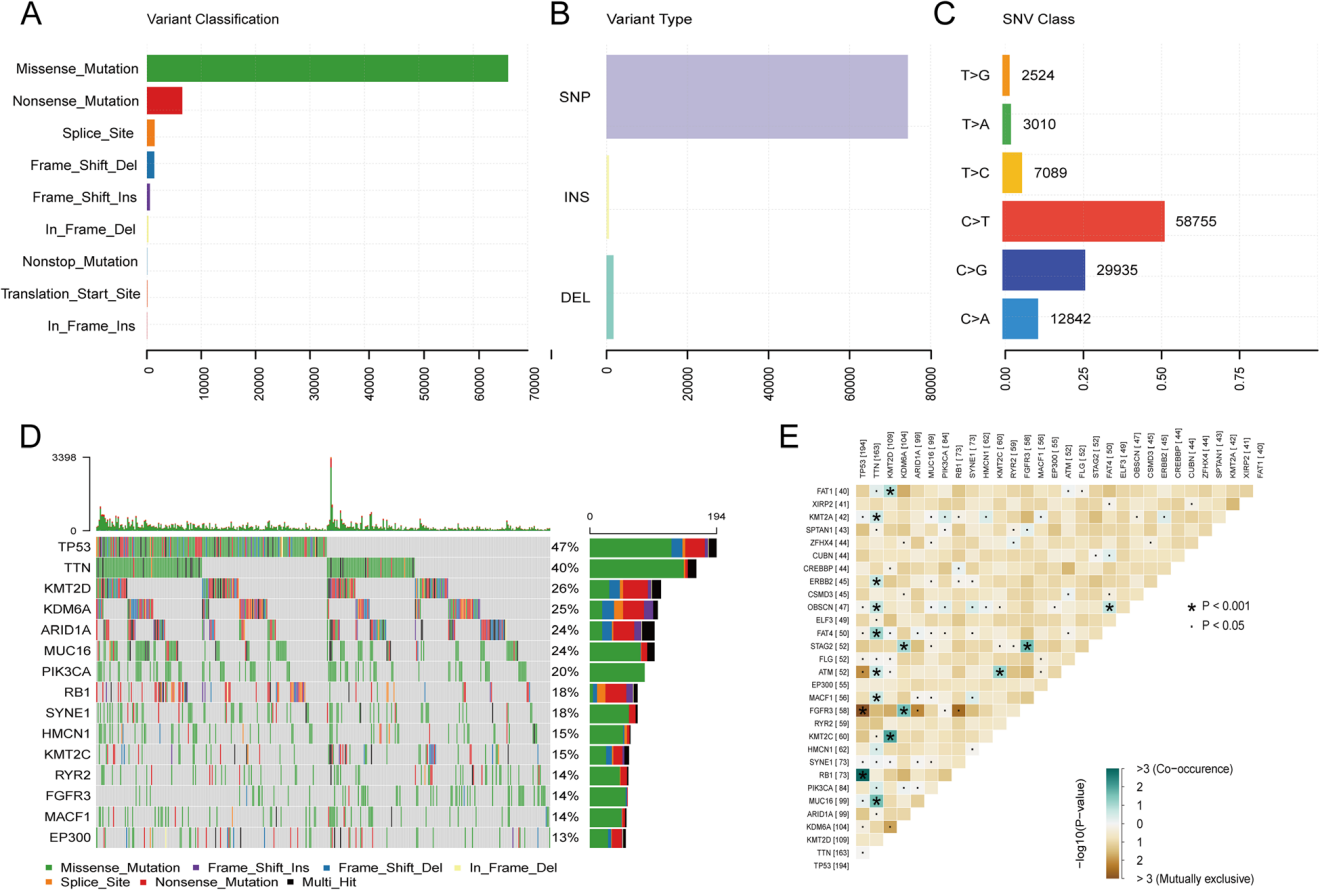
Overview of mutation information in BLCA patients. **a**-**c** Variant classifications, variant types and SNV classes in BLCA samples. SNP: single nucleotide polymorphism, INS: insertion, DEL: deletion, SNV: single nucleotide variants. **d** Waterfall plot showing the mutation information for top 15 genes. **e** The coincident and exclusive associations across the top 30 mutated genes

We analyzed and visualized the somatic mutation information of BLCA patients using the “maftools” package. Among all the mutation types, missense mutation is the most common, with a frequency far higher than that of other types (Fig. 1a). Single nucleotide polymorphism (SNP) represented the largest fraction in the variant type than insertion or deletion (Fig. 1b). The most common single nucleotide variant (SNV) was C > T, followed by C > G and C > A (Fig. 1c). The waterfall plot showed the top 15 most frequently mutated genes (Fig. 1d). Figure 1e shows the cooccurrence and exclusive associations between mutated genes, in which TP53 was tightly associated with FGFR3 and RB1.

### Clinical relevance and pathway analysis for TMB

**Fig. 2:**
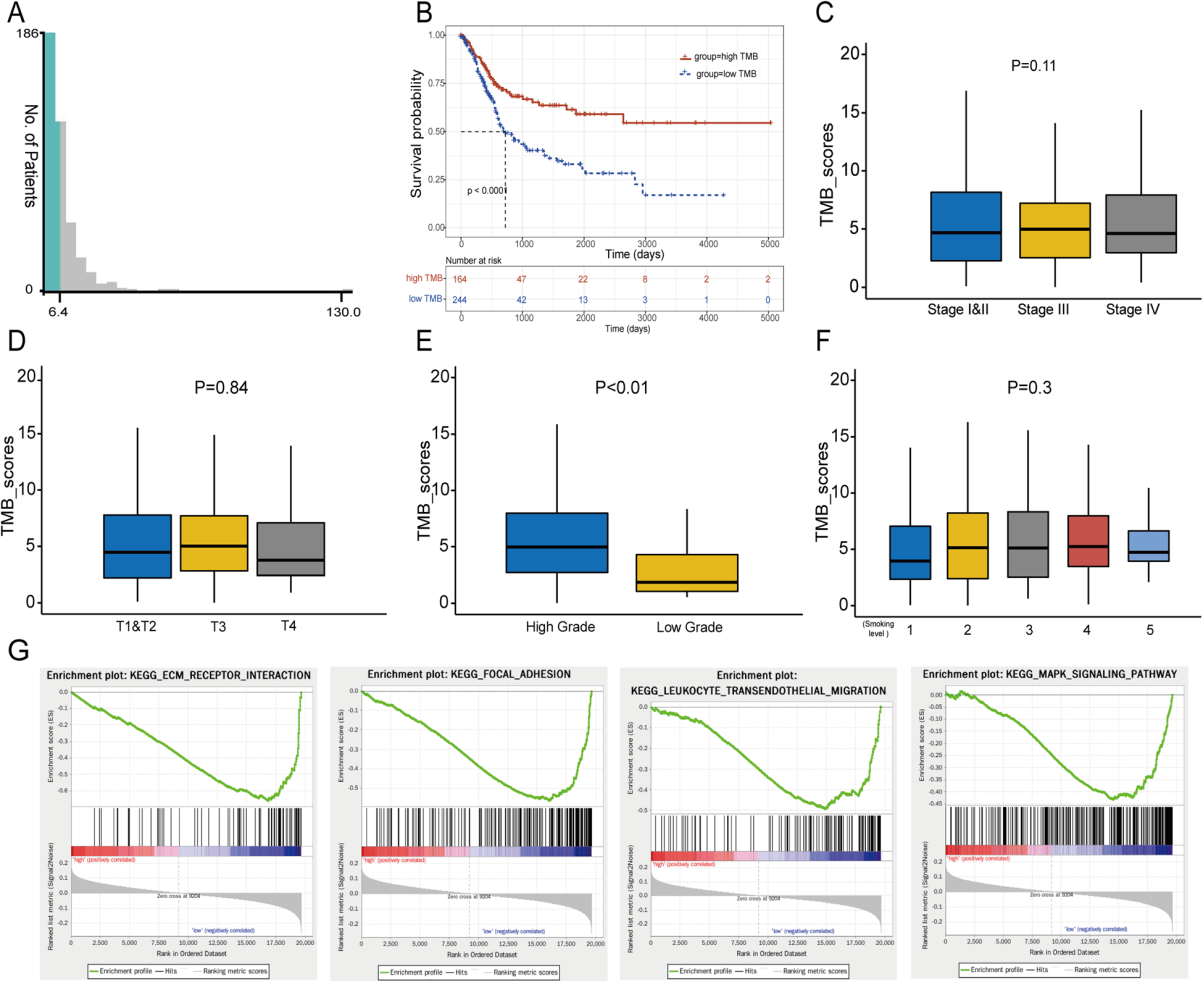
Clinical relevance and pathways analysis for TMB. **a** The best cut-off value of TMB was 6.4 computed by X-title. **b** KM curves of overall survival of high and low TMB groups. **c**-**f** The correlation between TMB scores and clinical stage, T stage, pathology grade and smoking level. **g** The top 4 results of GSEA between high and low TMB patients

The TMB score of each BLCA patient was calculated and the best cut-off value of TMB was 6.4 computed by X-title software (Fig. 2a). Then we divided the patients into a high TMB group with 164 cases and a low TMB group with 244 cases based on this cut-off. The result of KM survival analysis showed that the overall survival of the high TMB group was significantly better than the low TMB group (Fig. 2b, log-rank *P* < 0.0001). Clinical correlation analysis revealed that high-grade advanced tumors had higher TMB scores than low-grade tumors (Fig. 2e, *P* < 0.01). However, no significant associations were found between TMB scores and clinical stage, T stage, or smoking level (Figs. 2c, d and f).

In addition, GSEA was performed between high and low TMB groups to explore the biological pathways that may be affected by TMB. The results showed that BLCA samples in the low TMB group were significantly enriched for 41 biological processes, and the following four biological processes with high normalized enrichment scores and large sizes of gene counts were selected, including ECM (extracellular matrix) receptor interaction, focal adhesion, leukocyte transendothelial migration and MAPK signaling pathway (Fig. 2g). But for the high TMB group, no significant enrichment was observed.

### Screening and identification of key DEGs between high and low TMB groups

**Fig. 3:**
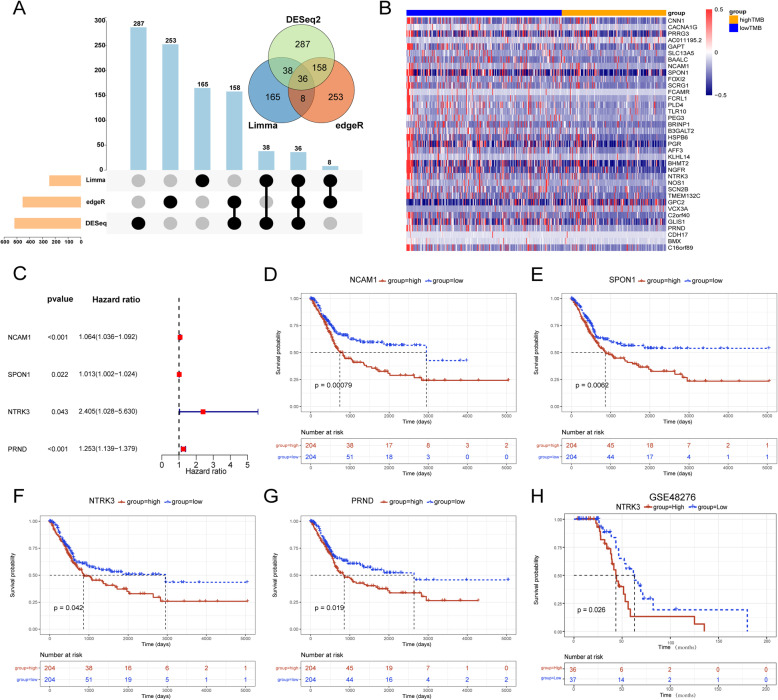
Screening of DEGs between high and low TMB groups associated with overall survival. **a** Upset plot and Venn plot of the results of three DEG-analysis methods. **b** The heatmap showed expression levels of 36 DEGs between high and low TMB groups. **c** The forest plot showed the results of univariate Cox analysis for 4 genes selected. **d**-**g** KM survival curves of 4 genes selected. (H) Validation of prognostic value for NTRK3 in GSE48276

Three methods were applied to screen for DEGs between high and low TMB groups. The intersection among the results of the three methods is presented in Venn diagram and Upset plot (Fig. 3a). Thirty-six genes were selected and the expression level of each gene in high and low TMB groups was shown in the form of heatmap (Fig. 3b). Univariate Cox analysis for each of the 36 genes showed that 10 genes were significantly related to overall survival (*P* < 0.05, Table 1). Then we performed KM survival analysis for these 10 genes and only four genes were with log-rank *P* < 0.05, including NCAM1, SPON1, NTRK3 and PRND (Table 1). The Forest plot showed the result of univariate Cox analysis for these genes (Fig. 3c). Figure 3d-g shows the KM survival curves for them. Among them, NTRK3 had the biggest hazard ratio (HR) value, indicating that the high expression of NTRK3 may be a risk factor. In addition, we validated the prognostic value of NTRK3 in another independent cohort (GSE48276, Fig. 3h). Therefore, we selected NTRK3 for further analysis.

**Table 1:** The 10 significant genes of univariate Cox analysis and their KM survival analysis results. (HR: Hazard Ratio; CI: Confidence Interval)

Gene	HR (95% CI)	Univariate cox ***P*** value	KM log-rank ***P*** value
PRRG3	1.876 (1.196–2.943)	0.006	0.142
SLC13A5	1.391 (1.109–1.744)	0.004	0.490
**NCAM1**	1.064 (1.036–1.092)	< 0.001	0.001
**SPON1**	1.013 (1.002–1.024)	0.022	0.006
B3GALT2	1.745 (1.384–2.200)	< 0.001	0.285
PGR	3.845 (1.560–9.478)	0.003	0.079
**NTRK3**	2.405 (1.028–5.630)	0.043	0.042
**PRND**	1.253 (1.139–1.379)	< 0.001	0.019
BMX	1.030 (1.017–1.042)	< 0.001	0.139
C16orf89	1.061 (1.007–1.118)	0.025	0.113

### NTRK3 coexpression in BLCA

**Fig. 4:**
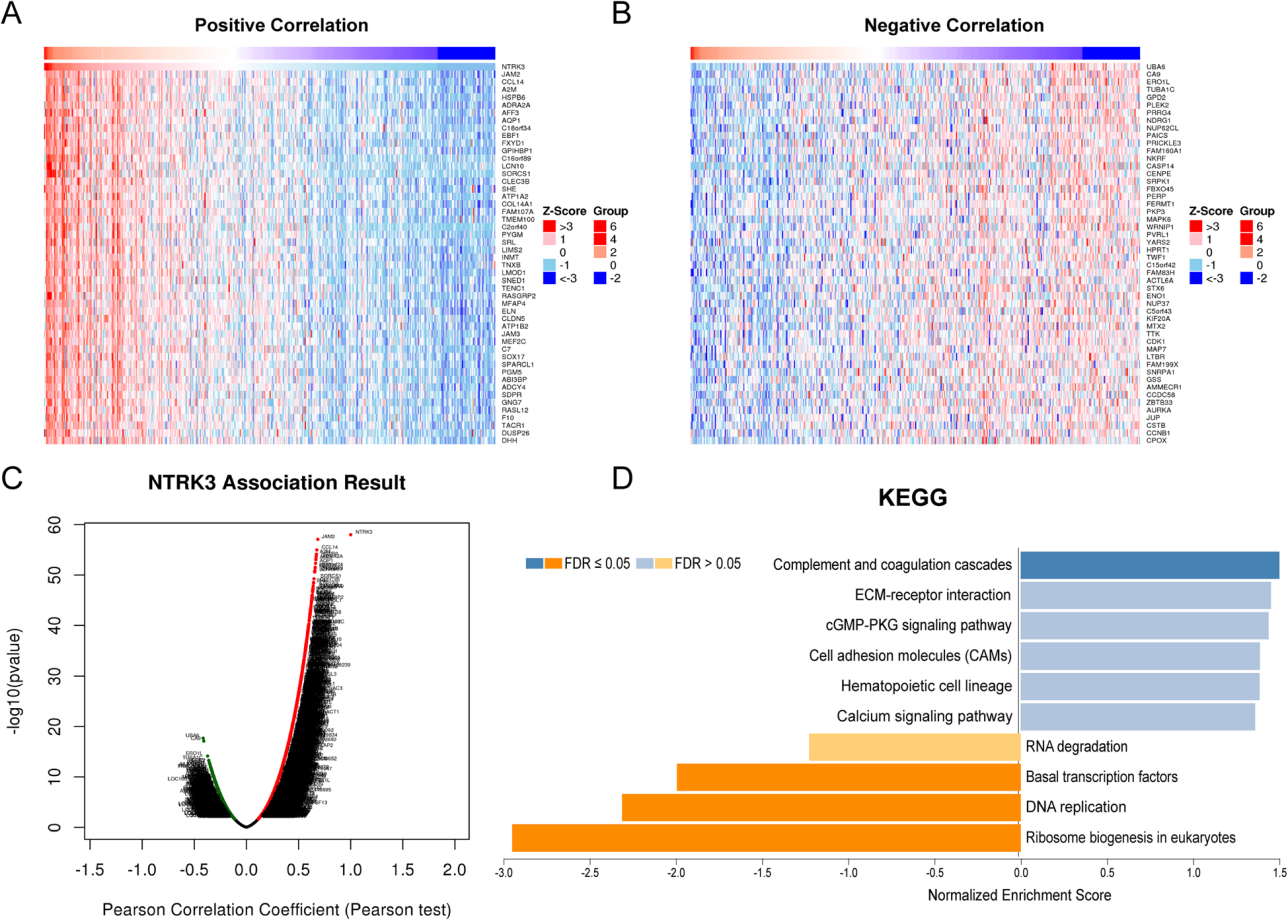
NTRK3 coexpression analysis in HCC (LinkedOmics). **a**, **b** Heat maps showing top 50 genes positively and negatively correlated with NTRK3 in BLCA. Red and blue represent positive and negative correlations, respectively. **c** The total NTRK3 highly correlated genes identified by Pearson test in BLCA cohort. **d** The significant KEGG pathways of NTRK3 in BLCA cohort

To explore the biological significance of NTRK3 in BLCA, the coexpression profiles of NTRK3 in the BLCA cohort were examined by LinkedOmics. As shown in Fig. 4C, 1,640 genes were significantly negatively correlated with NTRK3, and 5027 genes were significantly positively correlated with NTRK3 (FDR < 0.01). The top 50 significant genes positively and negatively correlated with NTRK3 are shown in heatmaps (Figs. 4a and b). GSEA enrichment results based on NTRK3 were dealt with “Weighted set cover” in LinkedOmics. The significant KEGG pathways identified included DNA replication, basal transcription factors, complement and coagulation cascades, ribosome biogenesis in eukaryotes (Fig. 4d).

### Relationship between TMB scores, NTRK3 expression level and ESTIMATE results

**Fig. 5:**
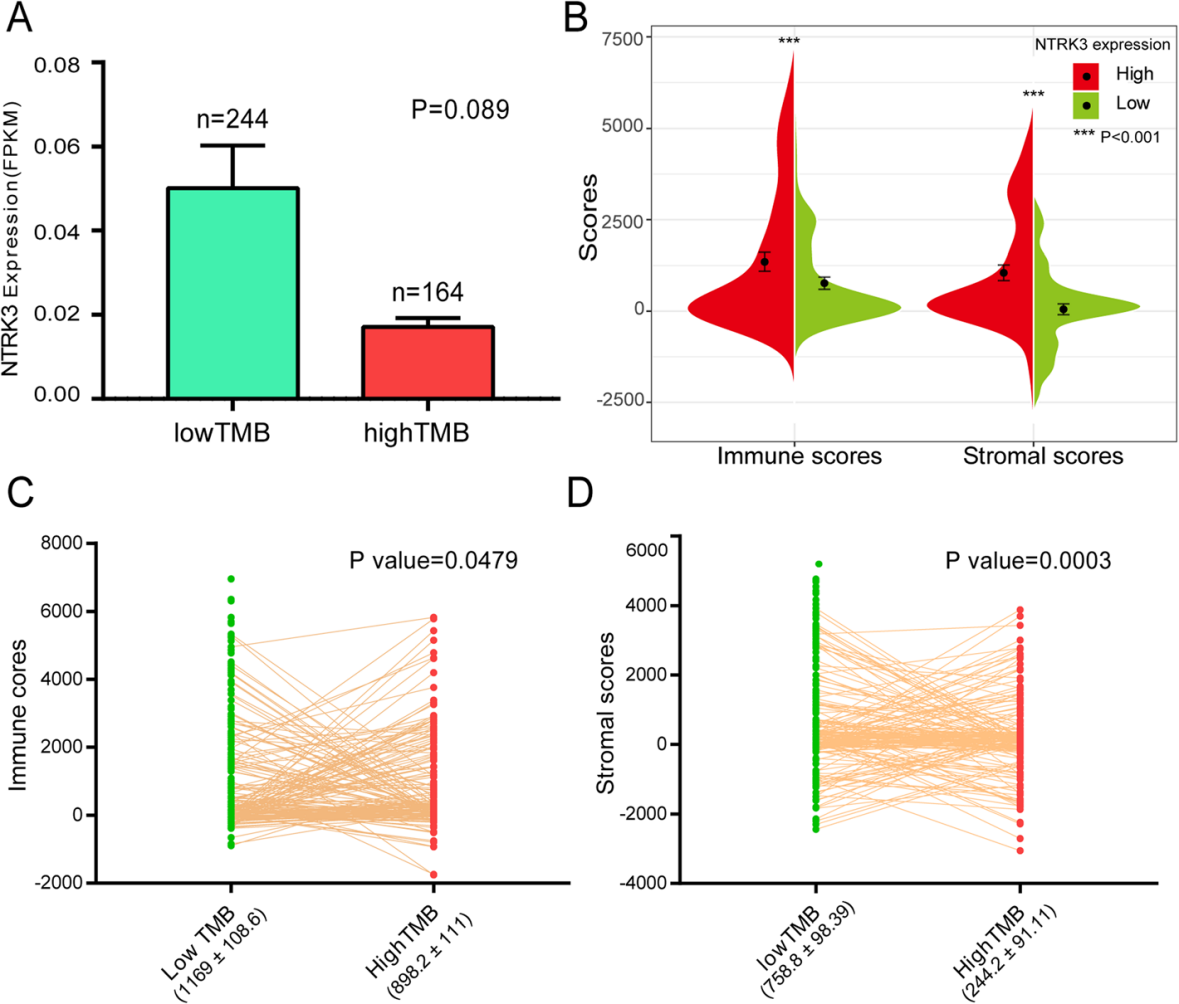
Relationship between TMB scores, NTRK3 expression level and ESTIMATE results. **a** Comparison of NTRK3 expression level between high and low TMB groups. **b** Comparison of immune and stromal scores between high and low groups based on NTRK3 expression. **c**, **d** Comparison of immune and stromal scores between high and low TMB groups

In the low TMB group, though the *P* > 0.05, the expression of NTRK3 was obviously higher than that of the high TMB group (Fig. 5a). Then, we calculated immune and stromal scores for the patients by ESTIMATE algorithm. In Fig. 5b, patients were divided into two groups according to the median value of NTRK3 expression. We found that the high expression group had significantly higher immune and stromal scores than the low expression group. Next, we measured the immune and stromal scores between high and low TMB groups. Compared with the high TMB group, the low TMB group had higher immune and stromal scores (Figs. 5c and d). All these results indicated that there was an evident correlation between TMB, NTRK3 and the infiltration level of immune cells and stromal cells. There may be a potential mechanism of interaction and mutual influence among them.

### Comparison of immune cell infiltration between high and low TMB groups and analysis of NTRK3 immune correlation

**Fig. 6:**
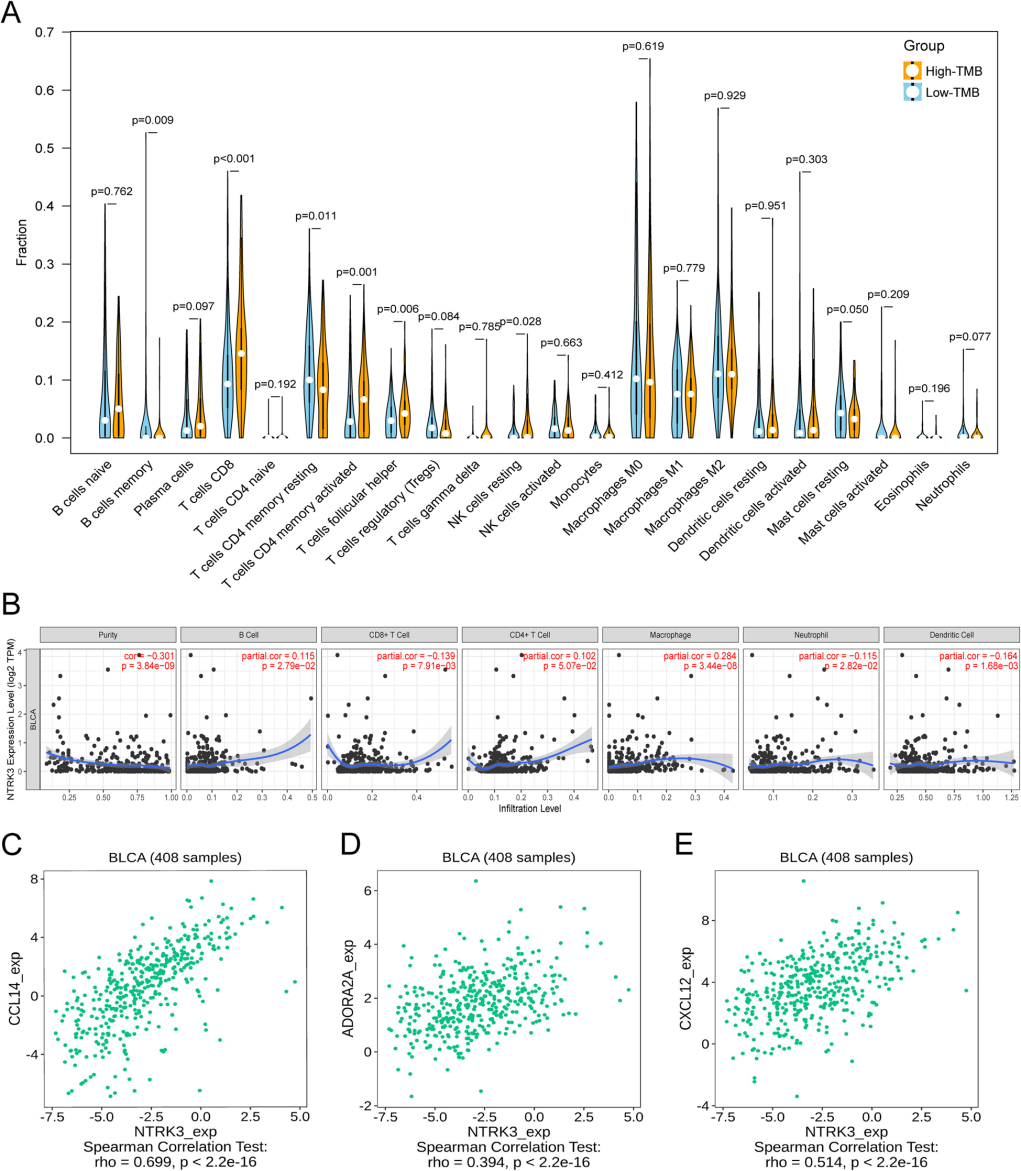
Immune infiltration analysis in BLCA. **a** Comparison of the infiltration of 22 leukocyte types between high and low TMB groups. **b** The correlation between NTRK3 and immune infiltrating cells. **c**-**e** The correlation between the expression of NTRK3 and the expression of CCL14, ADORA2A, and CXCL12 respectively

**Fig. 7:**
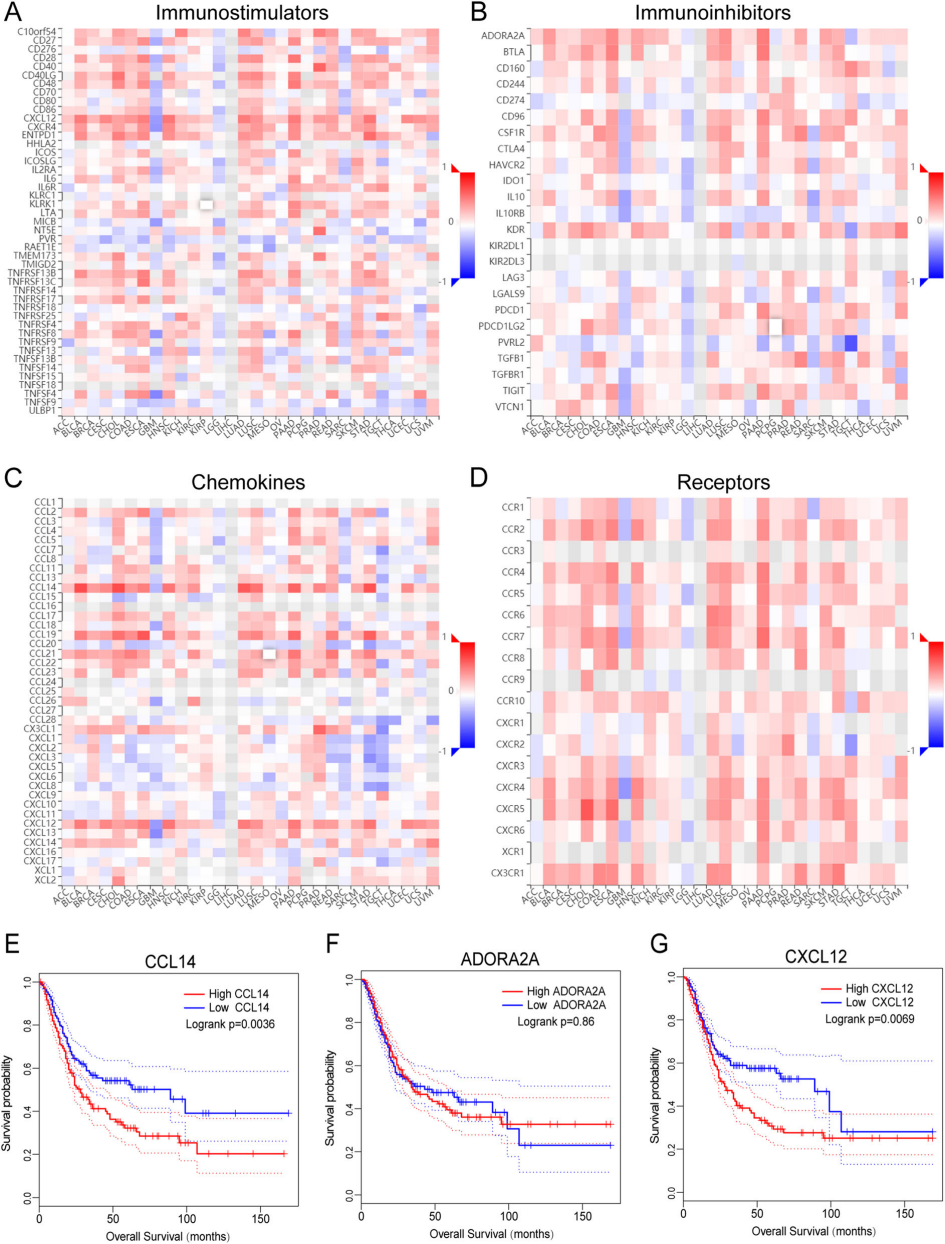
Relationship between NTRK3 expression and immunoregulators. **a**-**d** Spearman correlations between expression of NTRK3 and immunoregulators (Y axis) across human cancers (X axis). **e**-**g** The KM survival curves of CCL14, ADORA2A and CXCL12 in BLCA

Immune cell fractions for each sample were estimated by the CIBERSORT algorithm based on transcriptome data. After excluding samples with *P* values ≥0.05, 134 low-TMB samples and 102 high-TMB samples were selected for comparing fractions of 22 leukocyte subtypes between high and low TMB groups. The results showed that high-TMB samples had higher fractions of CD 8^+^ T cells, memory activated CD4^+^ T cells and T follicular helper cells (Tfh) while low-TMB samples had higher fractions of memory resting CD4^+^ T cells and resting mast cells (Fig. 6a). TIMER was used to assess the correlation between NTRK3 expression and common immune infiltrating cells. The expression level of NTRK3 was positively correlated with infiltrating levels of immune cells (B cell, CD8^+^ T cell, CD4^+^ T cell, macrophage, neutrophil, and dendritic cell) (Fig. 6b). Besides, we also evaluated the relationship between NTRK3 and immunomodulators through the TISIDB portal (Fig. 7a-d). The results showed that NTRK3 in BLCA has a significant positive correlation with chemokine CCL14, immunoinhibitor ADORA2A, and immunostimulator CXCL12 (Figs. 6c, d and e). Figure 7e-g shows the relationship between the three immunomodulators and overall survival in BLCA. Thus it can be seen that NTRK3 interacts with immune regulation in TME and may become a potential biomarker which has an important impact on the development of tumors and prognosis of patients.

### Validation for NTRK3 by Oncomine database and immunohistochemistry

**Fig. 8:**
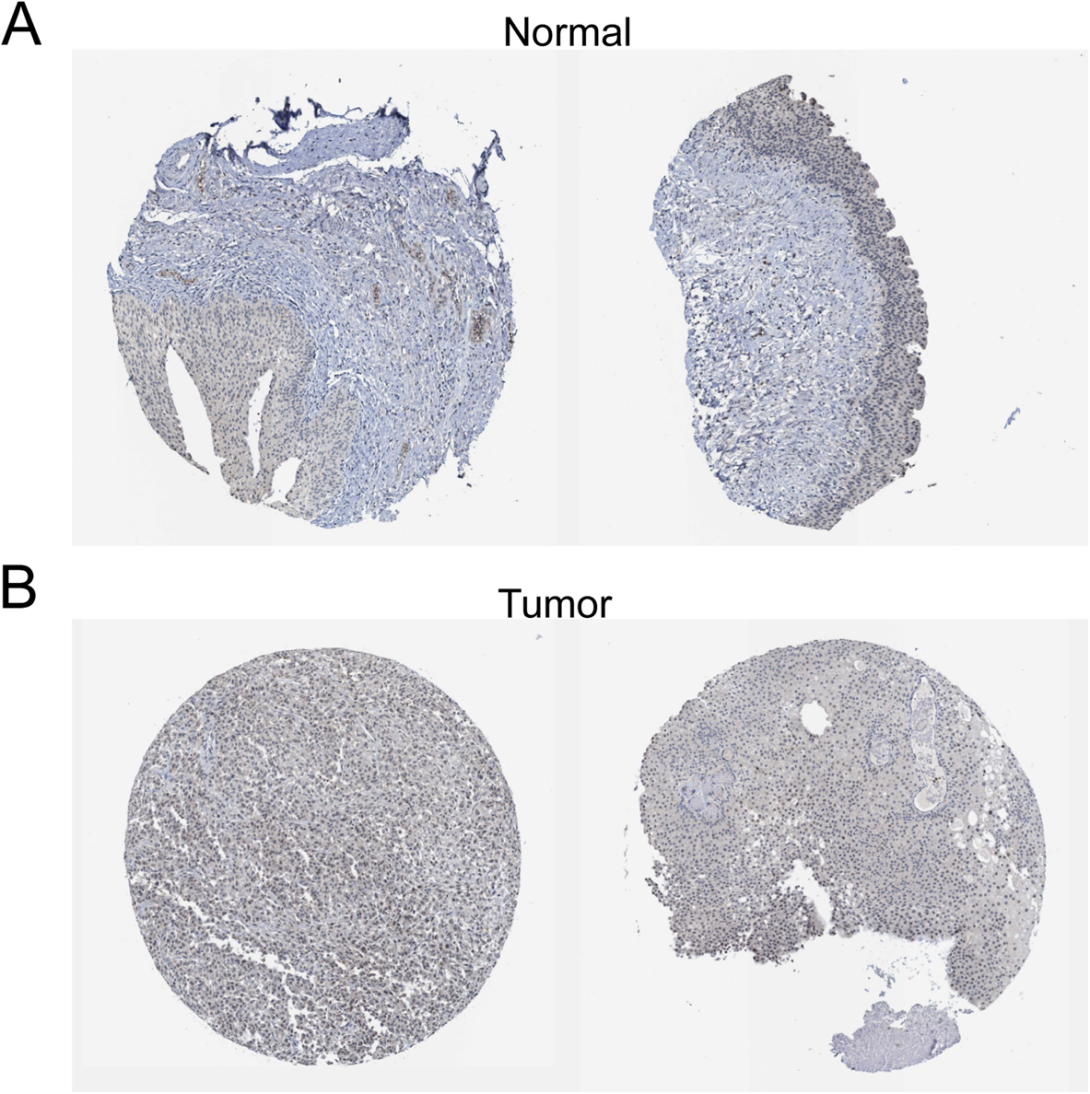
Immunohistochemistry of NTRK3 based on the Human Protein Atlas. **a** IHC staining of NTRK3 in bladder normal tissue. **b** IHC staining of NTRK3 in bladder tumor tissue

We searched the Oncomine database (https://www.oncomine.org) to compare the mRNA expression levels of NTRK3 between tumor and normal tissues. The result suggested that there was no significant difference between tumor and normal tissues in most studies (Supplementary Fig. S1). The immunohistochemistry data of NTRK3 was acquired from the Human Protein Atlas database (http://www.proteinatlas.org). As shown in Fig. 8, NTRK3 staining was higher in tumor samples than in normal tissue, which was consistent with the result of survival analysis, indicating that high expression of NTRK3 is a risk factor in bladder cancer.

## Discussion

Prior to the advent of ICIs, surgery and platinum-based systemic chemotherapy were the standards of care in BLCA. However, only 20% of MIBC patients are fit to receive chemotherapy and almost half of them may have sequelae with poor prognosis [24]. About 30–50% of metastatic MIBCs cannot receive cisplatin due to comorbidities, and until recently, platinum-based regimens were still the only treatment option, with no inspiring effect on clinical outcomes [25]. Nowadays, immunotherapy represented by ICIs has gradually revolutionized the treatment paradigm of BLCA. Indeed, the intravesical use of bacillus Calmette–Guérin (BCG) for treating NMIBCs has proven the immune response characteristic of BLCA since the 1970s [26]. Tumorigenesis is a process of mutation accumulation. BLCA has the third-highest level of somatic mutations and is highly antigenic which may facilitate the immunological recognition [27]. Consequently, it is crucial to fully understand and explore the role of TMB in BLCA immunotherapy.

In the present study, we found that BLCA patients with higher TMB had more survival benefits. This is consistent with the results of previous studies in other cancers such as cutaneous melanoma and ovarian carcinoma [28]. For patients treated with ICIs, high TMB has been confirmed to be associated with good response and better OS [29]. However, Cao et al. performed a pooled analysis of 103,078 patients to evaluate the predictive efficiency of TMB and found that the prognostic effect of TMB varied in patients with or without ICIs treatment, which might be related to tumor types [30]. Another research also indicated that the patients without ICIs treatment in some tumors may suffer a worse prognosis despite having high TMB [31]. Therefore, for the real application of TMB, it is still necessary to determine and assess its exact role in different tumors by more clinical trials. Moreover, meaningful GSEA results were all enriched in the low TMB group and most were immune-related, indicating that low TMB might enhance the tumor heterogeneity in BLCA. Of the GSEA results, ECM receptor interaction plays a crucial role in regulating invasiveness and progression of tumors. Cancer cells in the ECM of TME can release signals to mislead immune cells to avoid being attacked [32]. Besides ECM, the overactivation of focal adhesion pathway can result in dysfunction of cell migration and influence immune cell chemotaxis, which leads to tumor metastasis [33].

In terms of immune cell infiltration, the high proportion of CD4^+^ T cells, CD8^+^ T cells and Tfhs may be an important factor leading to better OS in the high TMB group. Tumor-infiltrating immune cells are a hallmark of immune surveillance and an integral part of complex microenvironment regulating tumor progression [34]. CD8^+^ T cells have strong cytotoxic activity for killing cancer cells, being considered as main drivers of anti-tumor immunity. The depletion in numbers and dysfunction of CD8^+^ T cells in TME create a favorable condition for cancer cell proliferation and metastasis [35]. CD4^+^ T cells and Tfhs also play a prominent role in anti-tumor immunity. CD4^+^ T cells can directly eliminate tumor cells through cytolysis or indirectly regulate the TME to target tumor cells [36]. And Tfh may conduce to the formation of tertiary lymphoid structures in primary site and thereby promote intertumoral immune response of CD8^+^ T cells and B cells [35]. ICIs can help restore T lymphocyte activity and break through the physical barrier of TME to promote T cell homing, thus activating anti-tumor immunity and improving the effect of immunotherapy [37].

After strict screening, NTRK3 was finally selected as a potential biomarker due to its excellent prognostic value and immune infiltration correlation. NTRK3 encodes the TRKC protein, a member of neurotrophic tropomyosin receptor kinase (TRK) family which regulates many aspects of neuronal development and function. After binding to the ligand neurotrophin-3 (NT-3), TRKC autophosphorylates and motivates various signaling pathways such as MAPK and PI3K/AKT pathways which can regulate cellular growth and differentiation [38]. In recent years, many studies have reported that TRK pathway aberrations such as single nucleotide variation, gene fusion and gene overexpression are involved in the pathogenesis of many cancers, among which NTRK3 gene fusion is the most fully verified carcinogenic event [39]. Except for the functional relevance of TRKC in the nervous system, the overexpression of TRKC is observed in many types of tumors, including neuroblastoma, breast cancer, hepatocellular carcinoma and metastatic melanoma. TRKC plays an important role in regulating angiogenesis, inducing tumor growth, preventing apoptosis and promoting metastasis. Abnormal activation of NTRK3 and its fusion proteins may regulate the epithelial-mesenchymal transition (EMT) process, tumor growth rate and tumorigenicity by activating several signaling pathways [40].

In our results, the expression level of NTRK3 was significantly positively correlated with the patients’ immune and stromal scores, suggesting that NTRK3 may be inherently regulated by the TME. Moreover, NTRK3 also showed good correlation with a variety of immune lymphocytes. Among the related immunomodulators, we were particularly interested in CCL14 and CXCL12, both of which were strongly positively coexpressed with NTRK3 and had prognostic value. CCL14 represents a C-C type chemokine with a high concentration in human plasma, mainly involved in the transport of lymphocytes and inflammatory cells [41]. The role of CCL14 in tumor progression is unclear and underreported, especially in bladder cancer. Yu et al. found that high expression of CCL14 played a protective role which can promote apoptosis of cancer cells and improve survival time in hepatic carcinoma [42]. However, Li et al. found that inhibiting the expression of CCL14 could effectively suppress the metastatic potential and angiogenesis of breast cancer [43]. In addition, there is an interesting theory that tumors can actively release chemokines to regulate the microenvironment and alter the host immune response from immunogenic to tolerogenic, thereby achieving immune escape and promoting tumorigenesis and metastasis. Shields et al. expounded this view in their research on CCL21 [44]. Thus, the underlying mechanism of the interaction between CCL14 and NTRK3 and how they jointly affect the progression of BLCA requires further investigation. As for CXCL12, it has been extensively studied in the field of cancers. CXCL12, also known as stromal cell derived factor-1 (SDF-1), was first characterized as a pre-B cell growth factor and combines its receptor CXCR4 as a signaling axis which mainly participated in many physiological processes such as hematopoiesis, immune responses and vascular formation [45]. Studies have shown that cancer cells in TME can induce overexpression of CXCL12 by autocrine or paracrine, and then activate downstream pathways to affect immune status and promote cancer cell proliferation and distant metastasis. Blocking the CXCL12/CXCR4 signaling axis may inhibit tumor growth and provide new ideas for immunotherapy [46]. Many biology processes in cancers are regulated by adenosine which exerts its control on both tumor and immune cells, modulating the main characteristics such as proliferation, metastasis and immune escape. A2A adenosine subtype controlled by ADORA2A is mainly present on the cell membrane of lymphocytes and able to reduce the activation of the machinery triggered by T cell receptor in immune cells, thus inducing a series of immunosuppressive events and promoting progression in tumors [47]. Cekic et al. found that A2A receptor (A2AR) regulated CD8^+^ T cells in TME and the application of its inhibitors could enhance the effect of immunotherapy in melanoma and bladder cancer cells [48]. A2AR antagonist showed monotherapy activity in a clinical trial in which patients were almost resistant or refractory to anti-PD (L)1 antibodies [49].

The current study has several limitations. First, our study is retrospective based on public databases and problems such as insufficient and limited data are inevitable. Second, the relationship between TMB and the response to ICIs cannot be evaluated in BLCA due to a lack of immunotherapy information of patients. Third, our results need to be verified in other independent patient cohorts with mutation information and by laboratory or clinical experiments.

## Conclusion

In conclusion, the present study systematically explored the relationship between TMB and prognosis as well as immune infiltration in BLCA. With further exploration, our data revealed that the expression of NTRK3 was closely correlated with survival and immune response. Accordingly, NTRK3 may prove to be a novel TMB-related biomarker and contribute to the development of immunotherapy for bladder cancer.

## Supplementary Information

**Additional file 1: Fig. S1**. Comparison of NTRK3 mRNA expression between tumor and normal tissues across multi-studies by Oncomine.

## Data Availability

All the data in our study can be accessed from the online public database and all the hyperlinks to publicly archived datasets were listed in the “Methods” part.

## Abbreviations

BLCA: Bladder cancer
TMB: Tumor mutation burden
TCGA: The cancer genome atlas
GSEA: Gene set enrichment analysis
DEGs: Differentially expressed genes
NMIBC: Nonmuscle-invasive bladder cancer
MIBC: Muscle-invasive bladder cancer
ICI: Immune checkpoint inhibitor
TME: Tumor microenvironment
FDR: False discovery rate
KEGG: Kyoto encyclopedia of genes and genomes
SNP: Single nucleotide polymorphism
SNV: Single nucleotide variant
OS: Overall survival
ECM: Extracellular matrix
NTRK3: NT-3 growth factor receptor
