# Supplementary figures and images for: Identification of NTRK3 as a potential prognostic biomarker associated with tumor mutation burden and immune infiltration in bladder cancer
Source: BMC Cancer. 2021 Apr 24;21:458. doi: 10.1186/s12885-021-08229-1 (PMC8070296; doi:10.1186/s12885-021-08229-1)

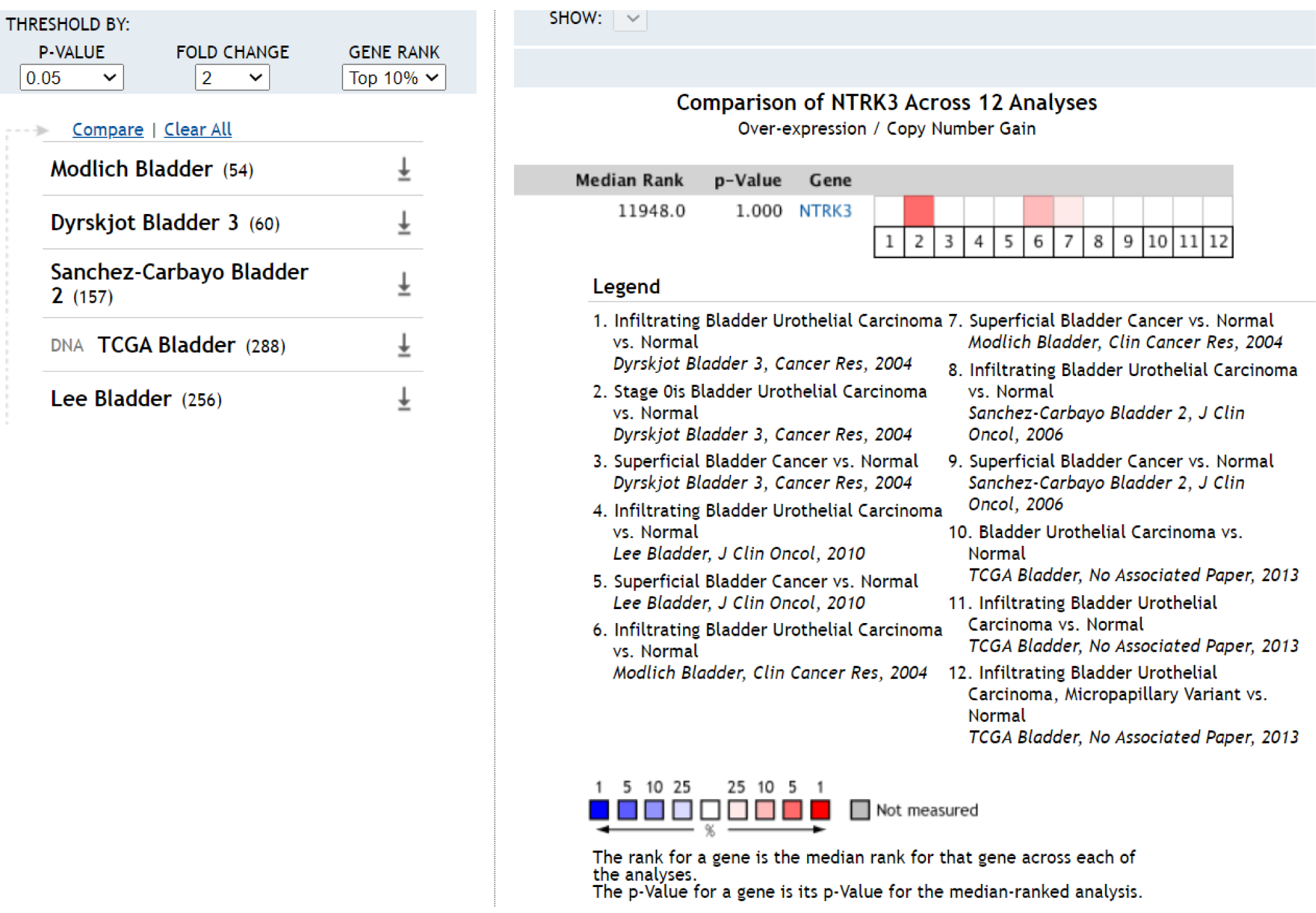

Supplement: Supplementary file 1 — Additional file 1: Fig. S1. Comparison of NTRK3 mRNA expression between tumor and normal tissues across multi-studies by Oncomine. [file 12885_2021_8229_MOESM1_ESM.tif]
